# Multilevel Modeling Analysis of Odontogenic Risk Factors and Nasal Septum Deviation Associated with Maxillary Sinus Mucosal Thickening: A Cone-Beam Computed Tomography Study

**DOI:** 10.3390/dj12030074

**Published:** 2024-03-13

**Authors:** Marwa Madi, Sara S. Alsaad, Nada AlAssiry, Dina Attia, Mansour AlAssiry, Osama Zakaria

**Affiliations:** 1 Department of Preventive Dental Sciences, College of Dentistry, Imam Abdulrahman Bin Faisal University, P.O. Box 1982, Dammam 31441, Saudi Arabia; saraalsaad22@outlook.com (S.S.A.); nada.mansour.a@hotmail.com (N.A.); 2 Department of Pediatric Dentistry and Dental Public Health, Faculty of Dentistry, Alexandria University, Alexandria 21527, Egypt; dina.ali@alexu.edu.eg; 3 Department of Otolaryngology, King Fahad Specialist Hospital, Dammam 32253, Saudi Arabia; mansour.alassiry@gmail.com; 4 Department of Biomedical Dental Science, College of Dentistry, Imam Abdulrahman Bin Faisal University, P.O. Box 1982, Dammam 31441, Saudi Arabia; oazakaria@iau.edu.sa

**Keywords:** maxillary sinus, posterior teeth, mucosal thickening, cone beam, computerized tomography, nasal septum

## Abstract

(1) Background: In this study, the impact of odontogenic risk factors with nasal septum deviation on maxillary sinus mucosal thickening was assessed using Cone-beam computed tomography CBCT. (2) Methods: A total of 328 maxillary sinus regions from 164 patients (85 males and 79 females) were examined. Images were interpreted by dental specialists and Otolaryngologists. Coronal and sagittal sections were examined to assess the proximity of the root tips of posterior maxillary teeth (RPMT) to the maxillary sinus. The periodontal bone loss for all maxillary posterior teeth was also assessed. Consequently, maxillary sinus mucosal thickening (MT) was further classified into three gradings. Multilevel modeling regression analysis was used due to the hierarchical structuring of the data. Four models were developed, a null model with no factors, a model with tooth-level factors (RPMT, PBL, tooth condition, and root length), a model with patient-level factors (gender and nasal septum deviation), and a model with combined patient- and tooth-level factors. Regression estimates (AOR) and 95% confidence intervals (CIs) of individual and tooth factors were calculated. (3) Results: Multilevel regression analysis showed that RPMT was significantly associated with MT of maxillary sinus (*p* < 0.001), where patients who had RPMT > 0 had higher odds of MT of maxillary sinus. Tooth condition was also found to be significantly associated with MT of maxillary sinus, where teeth with failed RCT (*p* < 0.001) and teeth with restorations (*p* < 0.008) had higher odds of MT of maxillary sinus (AOR = 2.87, 95%CI 1.65, 4.42, AOR = 1.64, 95%CI 1.14, 2.36, respectively). (4) Conclusions: In order to plan preoperative treatment for maxillary posterior teeth, it is important to assess the anatomical relationship between the sinus floor and the root tips of the maxillary posterior teeth. Additionally, we establish a better understanding of the clinician before surgical intervention is conducted.

## 1. Introduction

Mucosal thickness greater than 2 mm in the maxillary sinus mucous membrane is considered a pathological representation [1]. Maxillary sinus pathology may be of rhinogenic, odontogenic, traumatic, allergic, neoplastic, and bone-related origin [2]. Maxillary sinusitis is a frequent ear—nose—throat (ENT) infection [3]. Odontogenic sinusitis (ODS) is the infection of the maxillary sinus as a result of odontogenic disease [1]. Previous studies proposed certain conditions and procedures that provoke maxillary sinus infection, such as periodontal disease, trauma, apical periodontitis, extractions, endodontic treatment, and retained teeth [1,4]. The cause of mucosal thickening in the inferior aspect of the maxillary sinus was reported to be commonly due to dental pathology or intervention [5]. The proximity of the root apices to the maxillary sinus as well as the expansion of the sinus floor both contribute to an increased potential impact on the formation of odontogenic sinusitis [1].

Odontogenic sinusitis clinical presentation usually reveals a white emulsion-like purulent secretion in the middle meatus, nasal stinks, and radiographic imaging of unilateral opacifications of the maxillary sinus associated with dental lesions [2,3]. Various inflammatory and neoplastic conditions can present with unilateral sinus disease, which has a wider differential diagnosis than bilateral sinus disease [6]. Odontogenic sinusitis has been linked to unilateral maxillary sinusitis, but only a few studies have examined its prevalence among all sinusitis causes [6,7]. Endodontic and periodontic examinations, as well as cone-beam CT or peri-apical X-rays, are the dependable approaches used to confirm odontogenic disease [7]. 

Various clinical presentations could be encountered in patients with ODS, such as respiratory complaints, sinus congestion, isolated tooth pain, and asymptomatic radiographic abnormalities [6,8]. The literature confirms that sinus symptoms might precede or are concurrent with, or exhibit weeks, months, or years after dental symptoms occur. Therefore, a differential diagnosis of dental and non-dental causative factors is essential for precise management of sinusitis.

The incidence of ODS has increased from previous decades [9,10]. Currently, the literature reports that 10–12% and up to 14% of all sinusitis cases are related to odontogenic infections [9]. A high percentage of odontogenic sinusitis was evident with etiology related to unilateral sinus disease, comprising 45% of all cases, which was greater than all non-odontogenic inflammatory conditions combined [7]. The maximum incidences of odontogenic sinusitis were found mostly in the affected first and second upper molars, with a prevalence of 35.6% and 22% consecutively [9,11,12]. Previous CBCT studies [13,14,15] have reported that mucosal thickening greater than 2 mm was regarded as pathological alteration in the MS.

Radiographic imaging is crucial in identifying the odontogenic causes of ODS and complements clinical assessment findings [11]. CBCT has been preferred over traditional computed tomography (CT) due to its lower radiation exposure, cost-effectiveness as well as being a highly precise three-dimensional imaging technique [16]. Thus, it became a preferred non-invasive, quantitative method for examining both periodontal tissues and the maxillary sinus, thereby enhancing the probability of successful treatment outcomes [14]. It has been shown that CBCT imaging is crucial in the assessment of ODS, due to the exceptionally detailed envisioning of the hard tissues such as teeth, bones, and apical destructions and pathology—similarly in soft tissue imaging of the sinus in cases of mucosal thickening [12,13].

There is a growing recognition within the recent literature of odontogenic sinusitis’s role in sinus diseases. Research conducted by Alotibi et al. [17], Sendisci et al. [18], and Kaimal et al. [19] highlighted the link between dental pathologies, such as apical periodontitis and periodontal disease, and the onset of maxillary sinus mucosal thickening. These studies revealed that odontogenic sources significantly contribute to sinusitis cases, indicating an important area for in-depth exploration. Shanbhag et al. [20] observed significant associations between mucosal thickening greater than 2 mm and demographic factors like gender (males) and age (over 60 years), as well as dental conditions including periapical lesions and periodontal disease. These findings suggested that demographic and dental health factors together could play a crucial role in the development of maxillary sinus mucosal thickening.

Further, Troeltzsch [21] investigated potential risk factors and implications of residual disease following surgical intervention for odontogenic sinusitis. They emphasized the critical nature of addressing mucosal thickening to improve patient outcomes, advocating for a comprehensive approach to treatment.

These studies not only endorse the interplay between dental health and sinus diseases but also highlighted the essentials for dental professionals to employ a multidisciplinary strategy for the accurate diagnosis and management of odontogenic sinusitis. The synergy of dental assessments with advanced radiographic imaging, particularly CBCT, emerges as a cornerstone for enhancing diagnostic precision in ODS. Thus, the aim of the current study was to assess, by means of multilevel modeling analysis, odontogenic risk factors and nasal septum deviation on maxillary sinus mucosal thickening. The null hypothesis is that there is no relationship between posterior maxillary teeth and maxillary mucosal thickening.

## 2. Materials and Methods

### 2.1. Study Design

This retrospective cross-sectional study involves CBCT images taken for patients seeking dental care at COD IAU from January 2020 to March 2023. Ethical approval was obtained from the Imam Abdulrahman Bin Faisal University (IAU) Institutional review board (IRB-2022-02-307). The current study was conducted in accordance with the Strengthening the Reporting of Observational Studies in Epidemiology (STROBE) guidelines [22]. Written informed consent was obtained from all patients for the use of their cone-beam computed tomography (CBCT) images for research purposes. This consent included the understanding that their CBCT images might be used in future studies while ensuring patient confidentiality and data anonymization in compliance with ethical standards.

Inclusion criteria were adult patients of both genders, aged between 20 and 50 years, who were non-smokers and presented to the dental clinics for procedures like tooth extraction, endodontic treatment, implant placement, and orthodontic treatment. Exclusion criteria were subjects below 20 years of age, those with systemic diseases, or on medications influencing bone turnover. Incomplete patient records, skeletal asymmetries, partial CBCT scans, and the presence of artifacts or bone pathology also led to exclusion.

### 2.2. CBCT Scanning Procedure

The CBCT scans were conducted using the KODAK 9500 Cone-Beam 3D System (Carestream, Rochester, NY, USA), equipped with a flat panel detector. The scanned area was a cylindrical shape, with a height of 15–20.6 cm and a diameter of 9–18 cm, captured in the standard resolution mode with a voxel size of 0.2 mm. The machine operated at 90 kV tube voltage, 10 mA tube current, and an exposure time of 10.8 s. The CS 3D Imaging software (3.4.3. Carestream Health Inc., Atlanta, GA, USA) was employed for evaluating the obtained DICOM from the CBCT, and full volumes were thoroughly examined.

**Figure 1:**
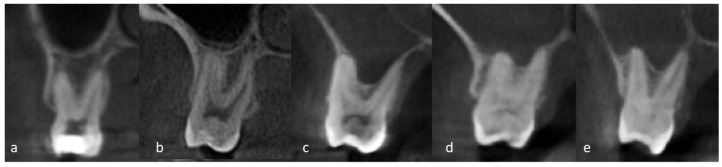
CBCT images showing proximity of the root tips of posterior maxillary teeth (RPMT) to the sinus floor. (**a**) Type 0: no contact with the cortical borders of the sinus. (**b**) Type 1: the root tips are in contact with the cortical borders of the sinus with an inferiorly curving sinus floor. (**c**) Type 2: the root tips are projecting laterally on the sinus cavity, but the apex is outside the sinus boundaries with an inferiorly curving sinus floor. (**d**) Type 3: the root tips are projecting into the sinus cavity with an inferiorly curving sinus floor. (**e**) Type 4: a superiorly curving sinus floor enveloping part or all of root tips.

The coronal and sagittal sections of the CBCT images were examined to assess the proximity of the root tips of posterior maxillary teeth (RPMT) to the sinus floor and were classified according to Sharan et al. [23] (Figure 1). Type 0: The RPMT is not in contact with the cortical borders of the sinus. Type 1: The RPMT is in contact with the cortical borders of the sinus with an inferiorly curving sinus floor. Type 2: The RPMT is projecting laterally on the sinus cavity, but its apex is outside the sinus boundaries with an inferiorly curving sinus floor. Type 3: The apex of RPMT is projecting into the sinus cavity with an inferiorly curving sinus floor. Type 4: A superiorly curving sinus floor enveloping part or all of RPMT.

**Figure 2:**
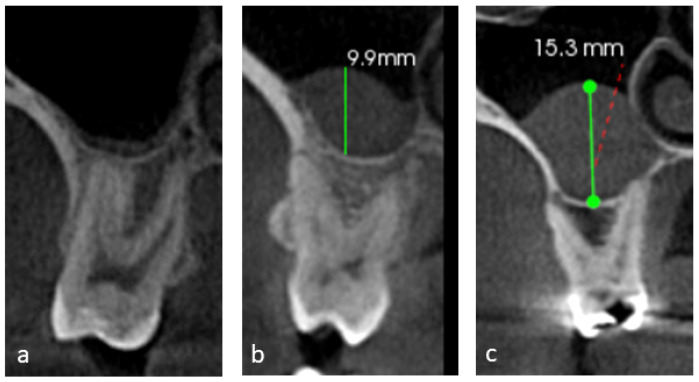
Coronal sections of CBCT images showing mucosal thickening grades. (**a**) Grade 1: 0–2 mm, (**b**) grade 2: 2–10 mm, and (**c**) grade 3: more than 10 mm.

The mucosal thickening of the maxillary sinus was measured on the coronal sections of the CBCT images at the point of maximum thickness from the sinus floor. The mucosal thickening was then classified [13] into 3 grades (Figure 2): grade 1 indicates 0–2 mm (considered within normal limits), grade 2 ranges from 2 to 10 mm (indicating moderate thickening), and grade 3 exceeds 10 mm (signifying severe thickening). Additionally, the assessment of periodontal bone loss for all maxillary posterior teeth followed the criteria set by Engebretson et al. [24], classifying the loss as Type 1 for normal to mild (<25% loss), Type 2 for moderate (25% to 50% loss), and Type 3 for severe (>50% loss).

Radiographic bone loss was measured on all teeth from 2 mm below the cement-enamel junction (CEJ) to the alveolar crest. This was followed by determining the length of the root from the CEJ to the root tip for each tooth. The bone loss percentage per tooth was then calculated by subtracting 2 mm from the distance between the alveolar crest and the CEJ, dividing this value by the root length, and multiplying by 100. To compute the overall bone loss for a subject, the percentages of bone loss for all teeth were summed and then divided by the total number of teeth examined.

To assess data reliability, intra- and inter-examiner calibrations were performed on 10 randomly selected CBCT scans. Three precalibrated examiners underwent a training session to familiarize themselves with the assessment criteria and protocols, followed by a testing phase, where their findings were compared to assess the inter-examiner agreement level. Every examiner repeated the measurements after 10 days to assess the intra-examiner agreement level. Agreement level was assessed using Cohen’s kappa coefficient. During CBCT assessment, discrepancies were discussed and resolved through consensus among the examiners.

**Figure 3:**
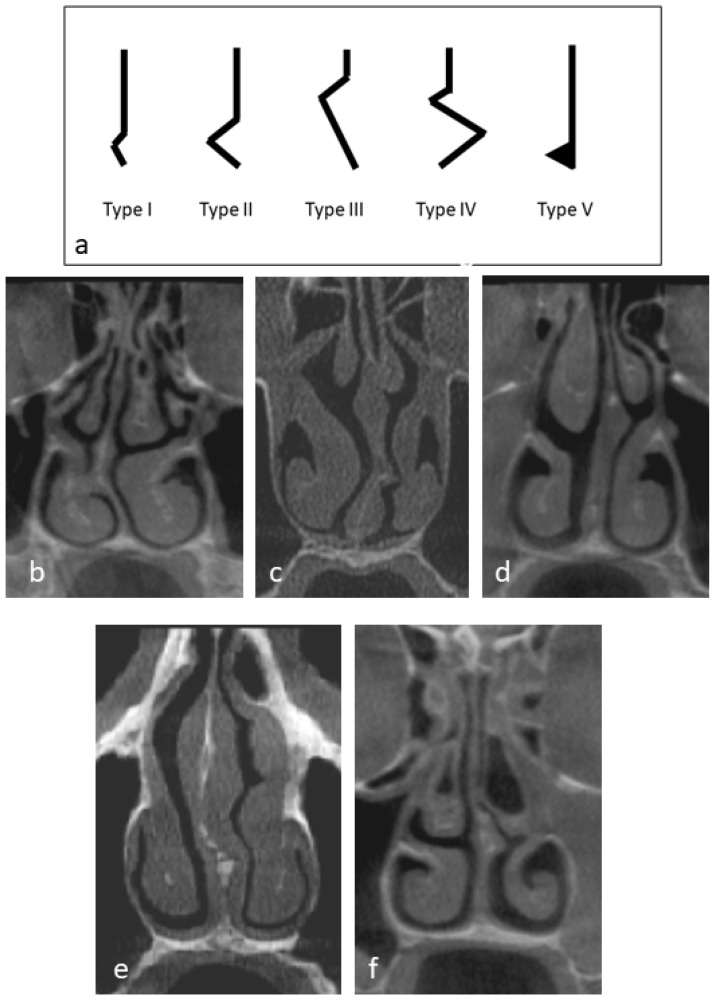
(**a**) Diagrammatic representation of nasal septum deviation types. Coronal cone-beam computed tomography of the patient demonstrating, (**b**) type I nasal septum deviation, (**c**) type II nasal septum deviation, (**d**) type III nasal septum deviation, (**e**) type IV nasal septum deviation, and (**f**) type V nasal septum deviation.

A precalibrated expert head and neck surgeon assessed the bony part of the nasal septum NS by examining continuous coronal sections of each CBCT scan, from the anterior nasal spine to the posterior nasal spine. Calibration was conducted by classifying the NS on randomly selected 10 CBCT scans and the examination was repeated after 10 days. Intra-examiner agreement level was calculated using Cohen’s kappa coefficient. The type of nasal septum deviation (NSD) was determined based on the most prominent view observed within the coronal cuts and classified according to Mladina et al. [25] (Figure 3).

### 2.3. Statistical Analysis

Data of this study were analyzed using the Statistical Package for the Social Sciences (SPSS), version 29.0 (IBM Corp., Armonk, NY, USA). Descriptive data were expressed as the percentage, mean, and standard deviation. Traditional statistical methods require individual independent variables, but the teeth observations in this study are not truly independent, since teeth are clustered inside patients. Consequently, multilevel binary logistic regression analysis was used to investigate the possible factors that could affect the mucosal thickening of maxillary sinus. First, an unconditional model was developed to assess if clustering was significant by producing an estimate of the intra-class correlation coefficient (ICC) using a model that contains no explanatory variables, the so-called intercept-only model or null model. Then, conditional models were developed by including fixed-effect factors with variance components covariance type. In step 2, fixed-effect variables for level 1 (tooth): The relation of the roots of posterior maxillary teeth to the sinus floor, root length, periodontal bone loss, and tooth condition. In step 3, level 1 fixed-effect factors were removed and replaced by level 2 (patient) factors: gender and nasal septum deviation. Finally, in step 4, both level 1 and level 2 factors were included. For the fully conditional model in step 4, we calculated the adjusted regression coefficient (AOR), 95% confidence intervals (CIs), and *p* values of all fixed-effect variables for levels 1 and 2. *p* values < 0.05 were considered statistically significant. For each model, pseudo R^2^ and percentage correctly classified were calculated to measure the model deviance. In addition, level 1 and 2 variances were calculated and used to calculate the ICC as a measure of clustering, where the ICC > 0.05 indicated substantial clustering at the patient level.

## 3. Results

The current study enrolled 169 patients who underwent CBCT, covering 328 maxillary sinuses (164 right and 164 left). The intra- and inter-examiner agreement levels were 97 and 95%, respectively, indicating a high level of consistency in their assessments. Table 1 shows that the sample was almost equally distributed in terms of gender (51.8% males), and in terms of not having nasal septum deviation (51.2%). For patients with nasal septum deviation, type III nasal septum classification was the most common (20.7%). Most of the investigated maxillary sinuses (79.9%) showed mucosal thickening, where almost half of the patients (46.3%) had bilateral MT of maxillary sinus.

A total of 1269 teeth were examined after excluding extracted teeth, 149 third molars, 289 molars, 279 first molars, 278 premolars, and 274 first premolars. In assessing MT in relation to the teeth examined, 499 teeth (39.3%) were associated with grade 2 and grade 3 (MT > 2 mm).

Table 2 shows the oral characteristics of examined teeth. Almost half the teeth examined were not in contact with the floor of the maxillary sinus (45.9%), vital (56.5%), and had a mean ± SD root length of 13.1 ± 2.4.

### Statistical Analysis

Model performance measures are specified in Table 3. It shows that there was clustering of teeth among patients where the ICC in the null model was >0.05. Pseudo R^2^ and percentage correctly classified for fixed effects of level 2 (patients-factors) were equal to the pseudo R^2^ and percentage correctly classified in the unconditional model, suggesting that majority of the explained variance was from level 1 (tooth-level) factors, rather than the level 2 (patient-level) characteristics.

Table 4 shows that the relation of the roots of posterior maxillary teeth (RPMT) to the sinus floor was significantly associated with MT of maxillary sinus, where patients who had RPMT > 0 had higher odds of MT of maxillary sinus (type 4 vs. type 0, AOR = 4.04, 95% CI 1.57, 2.86, type 3 vs. type 0, AOR = 3.15, 95% CI 1.52, 3.26, type 2 vs. type 0, AOR = 3.43, 95% CI 1.43, 4.08, type 1 vs. type 0, AOR = 2.80, 95% CI 1.98, 13.26).

Tooth condition was also found to be significantly associated with MT of maxillary sinus, where teeth with failed RCT and teeth with restorations had higher odds of MT of maxillary sinus (AOR = 2.87, 95%CI 1.65, 4.42, AOR = 1.64, 95%CI 1.14, 2.36, respectively). None of the patient-level factors were significantly associated with MT of maxillary sinus in addition to periodontal bone loss and root length from tooth-level factors.

## 4. Discussion

In this study, 328 maxillary sinuses (164 R and 164 L), and 1269 teeth, were examined by CBCT to assess the MT and the odontogenic risk factors that may influence it, together with the associated nasal septum condition. While the proximity of maxillary posterior teeth to the sinus is acknowledged, the distinctive contribution in the current study lies in the application of multilevel modeling analysis (MLM). The significance of MLM lies in its capacity to address hierarchical structures and nested data, enabling a comprehensive exploration of variability across different levels of analysis. The present study used variance component models to correctly specify the data structure and to analyze the risk factors of maxillary sinus mucosal thickening. The major findings of this study were that there was clustering of teeth within patients, and a significant amount of the variability in MT of maxillary sinus was explained by tooth factors rather than patient factors. As for the patient factors, there were higher odds of increased mucosal thickness in males compared to females, and in patients with nasal septum deviation, but these differences were not statistically significant. Regarding the tooth factors, the present findings showed that teeth with failed RCT and teeth with restorations had higher odds of MT of maxillary sinus.

The present findings are in agreement with those of Lu et al. [26], where they observed a notable increase in the thickening of the maxillary sinus mucosa correlating with an increase in the number of teeth affected by periapical lesions. This observation is further supported by research conducted by Sheikhi et al. [27,28] and Goller-Bulut et al. [29], which highlighted a significant impact of failed endodontic treatments and severe tooth decay on MT. The occurrence of this thickening in relation to teeth with inadequate endodontic treatment can be attributed to the potential penetration of endodontic instruments into the maxillary sinus during procedures. This penetration can introduce irrigating solutions, sealants, or filling materials into the sinus [30]. Additional studies [31,32] have indicated that infections spreading from periapical or periodontal diseases associated with the posterior teeth can escalate into sinusitis, as the infection extends beyond the dental tissue into the maxillary sinus.

Previous studies [33,34,35] that used cone-beam computed tomography to assess the alveolar dimensions of maxillary molars reported significant variances in the distances from the root apex to the sinus floor (SF), especially the pronounced intrusion of palatal roots into the SF. A previous report [36] showed that maxillary sinusitis was frequently observed in patients’ presenting with apical periodontitis in relation to the maxillary first molar. These observations are significant for risk assessment in immediate implant placement and endodontic procedures. Moreover, Wehrbein and Diedrich [37] observed a direct correlation between the extent of pneumatization post-tooth extraction and the length of root projecting into the maxillary sinus.

Our results showed a significant association between the proximity of the roots of posterior maxillary teeth (RPMT) to the sinus floor and mucosal thickening (MT) of the maxillary sinus. Patients with a RPMT greater than type 0 had increased chances of experiencing MT of the maxillary sinus. A toral of 45.9% of the examined teeth were not in contact with the cortical borders of the sinus, and 7.9% of the teeth had their roots penetrating into the MS. These results differ to those reported by Aguori et al. [15]; in their study about sound maxillary posterior teeth, they found that almost 50% (*n* = 81) of the examined MS had at least one posterior root apex protruding into it. However, no significant difference in the prevalence and average values of MT based on gender or root apex proximity types was detected [15]. This difference could be attributed to the differences in race or age, as well as the different diagnostic techniques.

In the present study, 40% of the examined teeth (499 teeth) showed MT more than 2 mm, this is in agreement with previous studies, in which the prevalence of mucosal thickening (MT) greater than 2 mm in patients without periapical lesions was remarkably high, ranging from 10.6% to 60.5% [13,15,26,27].

Our results indicated that males had greater odds of having increased mucosal thickness compared to females. This is in agreement with other studies that have shown a significant [13,14,27] or non-significant [38,39] higher prevalence of MT greater than 2 mm in males. Aguori et al. [15] observed a non-significant increased prevalence in females (42.7%) compared to males (29.2%).

Nurbakhsh et al. [40] also found comparable results, demonstrating that the greater the proximity of the tooth apex or periapical lesion to the MS, the greater the increase in sinus mucosal thickening (MT). Our findings indicate a significant association between the condition of teeth, especially those with failed RCT and restorations, and increased MT of the maxillary sinus. This is in agreement with previous studies [20,26,28,29] that have identified a higher risk of sinus infections linked to periapical lesions near or associated with the sinus floor, with larger lesions contributing to increased MT of the maxillary sinus.

In the current study, almost 50% of the patients had NSD and type III nasal septum classification was the most common (20.7%). Patients with NSD had 0.74 fold the odds (or 26% lower odds) of increased mucosal thickness of the maxillary sinus compared to those without deviation; however, this difference was not statistically significant. This is in agreement with previous studies [41,42,43] that observed sinus pathology, mucosal thickening and an increase in the maxillary sinus volume in most patients with NSD and obstruction even when nasal symptoms were absent. In contrast to our findings, Smith et al. [44] and Mohebbi et al. [45] reported that no correlation was identified between septal deviation and maxillary sinusitis. The discrepancy in findings between our study and those findings could be attributed to various factors, such as differences in the study design, sample size, demographic characteristics of the participants, or the methods used to assess and classify septal deviation and maxillary sinusitis. Additionally, variations in the severity and types of septal deviations considered, and the criteria used to diagnose maxillary sinusitis, could also contribute to the contrasting results.

In the current study, type III NSD was the most common (20.7%) observed among 85 patients with nasal septum deviation. In contrast, Shoib and Viswanatha [46] observed that out of their 200 observed cases, 50 patients with bilateral maxillary sinusitis (25%) were associated with an S-shaped deviated nasal septum, while 38 patients with unilateral maxillary sinusitis and 29 patients with bilateral maxillary sinusitis were associated with spur type of deviated nasal septum. Tassoker et al. [47] analyzed 110 CBCT images and found that the maxillary sinus volume (MSV) did not significantly relate to nasal septal deviation (NSD), concha bullosa (CB), impacted teeth, or age. However, they observed that gender played a significant role, with males having larger average MSVs compared to females.

A limitation of the current study is the relatively limited sample size, in which the findings may not be fully representative of the broader population. The reliance on CBCT, while offering detailed insights, is also subject to the inherent limitations of the technology, including resolution limits and potential artifacts. Including patients that have already been diagnosed with chronic maxillary sinusitis would have added more significance to the findings as well as assessing the influence of smoking on maxillary sinus mucosa.

Given the findings and limitations, several recommendations emerge. A larger and more diverse sample size, encompassing various age groups, ethnicities, and geographical locations, could offer more generalized and robust insights. The significant association of the relation of the roots of posterior maxillary teeth to the sinus floor with MT of the maxillary sinus underscores the need for enhanced clinical attention to dental health and interventions, especially involving the posterior maxillary teeth. Furthermore, the significant association of teeth with failed RCT and restorations with MT of maxillary sinus calls for improved quality and precision in endodontic and restorative practices. Collaborative research involving dental and maxillofacial specialists could unveil nuanced insights into the intricate relationships between dental health, anatomical structures, and maxillary sinus health.

## 5. Conclusions

This study reveals complex connections between the root of posterior maxillary teeth and the maxillary sinus floor. Deviated nasal septum and several odontogenic risk factors influence the health of the maxillary sinus. When planning treatment for maxillary sinus lesions, it is important to take into account the condition of the patient’s teeth and their periodontal health, as these factors can increase the risk of sinus infections. Collaborative clinical assessment between dental specialists and ORL is advocated in the case of abnormal representation of the maxillary sinus to ensure accurate diagnosis.

## Figures and Tables

**Table 1:** Sample characteristics.

			n	%
Gender (*n* = 164 patients)	Male		85	51.8
	Female		79	48.2
Nasal septum classification (*n* = 164)	No nasal septum deviation		84	51.2
	Type I		15	9.1
	Type II		16	9.8
	Type III		34	20.7
	Type IV		3	1.8
	Type V		12	7.3
Prevalence of MT (*n* = 164 patients)	Yes		131	79.9
	No		33	20.1
Distribution of MT (*n* = 131 patients)	Unilateral	Right	21	12.8
		Left	34	20.7
	Bilateral		76	46.3
Teeth classification (*n* = 1269 teeth)	#18 and 28		149	11.7
	#17 and 27		289	22.8
	#16 and 26		279	22.0
	#15 and 25		278	21.9
	#14 and 24		274	21.6
Classification of MT (*n* = 1269 teeth)	0–2		770	60.7
	2–10		313	24.7
	>10		186	14.7

**Table 2:** Oral characteristics of teeth examined (N = 1269 teeth).

		n	%
The relation of roots of posterior maxillary teeth to the sinus floor (RPMT)	Type 0	583	45.9
	Type 1	403	31.8
	Type 2	183	14.4
	Type 3	77	6.1
	Type 4	23	1.8
Radiographic bone loss	<25%	1148	90.5
	25–50%	68	5.4
	>50%	53	4.2
Teeth condition	Vital	717	56.5
	Restoration	317	25.0
	Failed RCT	156	12.3
	RCT	51	4.0
	Remaining root	15	1.2
	Implant	13	1.0
Root length (mean ± SD)		13.1 ± 2.4	

**Table 3:** Models specification and performance comparison.

	Unconditional Model	Model with Level 1 Fixed-Effect Factors	Model with Level 2 Fixed-Effect Factors	Model with Level 1 and Level 2 Fixed-Effect Factors Combined
ICC	0.32	0.36	0.32	0.33
Pseudo R^2^	0.27	0.34	0.27	0.35
Percentage correctly classified	76.4	79.3	76.4	79.6

ICC: intra-class correlation coefficient. Level 1 (tooth) fixed-effect factors: tooth condition, periodontal bone loss, root length, and Sharan classification. Level 2 (patient) fixed-effect factors: gender, nasal septum deviation.

**Table 4:** Association between patient-level factors and tooth-level factors and mucosal thickness of maxillary sinus.

		Mucosal Thickness of Maxillary Sinus Presence		
		AOR	95% CI	*p* Value
Patient-level factors				
Gender	Male	1.46	0.92, 2.33	0.13
	Female	Reference		
Nasal septum deviation	Yes	0.74	0.46, 1.20	0.24
	No	Reference		
Tooth-level factors				
The relation of roots of posterior maxillary teeth to the sinus floor (RPMT)	Type 1	2.80	1.98, 13.26	<0.001 *
	Type 2	3.43	1.43, 4.08	<0.001 *
	Type 3	3.15	1.52, 3.26	<0.001 *
	Type 4	4.04	1.57, 2.86	<0.018 *
	Type 0	Reference		
Periodontal bone loss	>50%	2.29	1.36, 4.82	0.07
	25–50%	1.34	1.02, 2.94	0.38
	<25%	Reference		
Tooth condition	RCT	1.43	0.68, 2.98	0.34
	Failed RCT	2.87	1.65, 4.42	<0.001 *
	Restoration	1.64	1.14, 2.36	0.008 *
	Implant	0.25	0.05, 1.35	1.12
	Remaining root	1.78	0.44, 7.01	0.42
	Vital	Reference		
Root length		0.96	0.91, 1.04	0.18

* p value less than 0.05.

## Data Availability

Data will be provided upon request from the corresponding author.
